# Do Impairments in Visual Functions Affect Skiing Performance?

**DOI:** 10.3389/fnins.2021.648648

**Published:** 2021-05-13

**Authors:** Amritha Stalin, Marieke Creese, Kristine Nicole Dalton

**Affiliations:** School of Optometry & Vision Science, University of Waterloo, Waterloo, ON, Canada

**Keywords:** contrast sensitivity, visual acuity, visual field, Paralympic alpine skiing, Paralympic nordic skiing

## Abstract

Nordic and alpine skiing-related visual tasks such as identifying hill contours, slope characteristics, and snow conditions increase demands on contrast processing and other visual functions. Prospective observational studies were conducted to assess the relationships between skiing performance and a broad range of visual functions in nordic and alpine skiers with vision impairments. The study hypothesized that contrast sensitivity (CS), visual acuity (VA), and visual field (VF) would be predictive of skiing performance. Binocular static VA, CS, light sensitivity, glare sensitivity, glare recovery, dynamic VA, translational and radial motion perception, and VF were assessed in elite Para nordic (*n* = 26) and Para alpine (*n* = 15) skiers. Skiing performance was assessed based on skiers’ raw race times. Performance on the visual function tests was compared with skiing performances using Kendall’s correlations (with and without Bonferroni–Holm corrections) and linear multivariable regressions (*p* < 0.05 considered significant). None of the vision variables were significantly correlated with performance in Para nordic or Para alpine skiing after Bonferroni–Holm corrections were applied. Before applying the corrections, VF extent (ρ = -0.37, *p* = 0.011), and static VA (ρ = 0.26, *p* = 0.066) demonstrated the strongest correlations with Para nordic skiing performance; in Para alpine skiing, static VA and CS demonstrated the strongest correlations with downhill (static VA: ρ = 0.54, *p* = 0.046, CS: ρ = -0.50, *p* = 0.06), super G (static VA: ρ = 0.50, *p* = 0.007, CS: ρ = -0.51, *p* = 0.017), and giant slalom (static VA: ρ = 0.57, *p* = 0.01, CS: ρ = -0.46, *p* = 0.017) performance. Dynamic VA and VF were significantly associated with downhill (ρ = 0.593, *p* = 0.04) and slalom (ρ = -0.49, *p* = 0.013) performances, respectively. Static VA was a significant predictor of giant slalom [(*F*(3,11) = 24.71, *p* < 0.001), and *R* of 0.87], super G [(*F*(3,9) = 17.34, *p* = 0.002), and *R* of 0.85], and slalom [(*F*(3,11) = 11.8, *p* = 0.002), and *R* of 0.80] performance, but CS and VF were not. Interestingly, static VA and CS were highly correlated in both Para nordic (ρ = -0.60, *p* < 0.001) and Para alpine (ρ = -0.80, *p* < 0.001) skiers. Of the vision variables, only static VA and VF were associated with skiing performance and should be included as the in Para nordic and Para alpine classifications. The strong correlations between static VA and CS in these skiers with vision impairment may have masked relationships between CS and skiing performance.

## Introduction

Sports and exercise play significant roles in improving the physical and mental health of individuals with visual disabilities (Gleeson et al., 2014; Koolaee, 2017; Fontenot et al., 2018). While the multidisciplinary rehabilitation strategies integrating sports with traditional methods assist at individual levels, international multi-sport events like Paralympics promote positive changes in societal attitudes toward individuals with disabilities and accelerate advancements in accessibility (Gold and Gold, 2007; Ferrara et al., 2015). Although athletes with visual impairment (VI) have been participating in the Paralympics since 1976 (Tweedy et al., 2014), research exploring the impact of vision impairment on sport performance has been limited.

Nordic skiing and alpine skiing are the only two VI sports in the Winter Paralympics (International Paralympic Committee, 2019). Being highly dynamic in nature, these sports demand rapid processing of visual information. Skiers must make quick decisions and vary their speed, direction, or body position based on their visual feedback (Decroix et al., 2017). Research has demonstrated that the major visual cues that elite, able-sighted alpine skiers rely on are the positions of gates on the course, their pole positions, and terrain cues such as the turn initiation and take-off points, the slope and curve of the hill, and distinctive holes and bumps and remarkable transitions on the course. Skiers also reported that the blue-colored markings on the left and right sides of the courses help them to orient, especially in the presence of fog or shadow (Schläppi et al., 2016). Although a similar study has not been done in nordic skiing, it is possible that some of the visual cues nordic skiers use are similar due to similarities in the sport environments.

Identifying the abovementioned visual cues would require skiers to have reasonable distance visual acuity (VA), contrast sensitivity (CS), depth perception, and peripheral vision while they are static, as well as while moving (Craybiel et al., 1955; Senner et al., 1999; Erickson, 2007). Senner et al. (1999) reported that a 20% decrease in static VA could significantly affect the reaction times of leisure skiers to smaller and low-contrast objects such as ice patches, even though their reaction times to larger obstacles such as standing or moving skiers were not affected. Furthermore, skiers’ motion and reduced visibility due to extrinsic factors related to weather, lighting, or snow conditions decrease the relative contrast of the visual information, increasing the demand on contrast processing (Erickson, 2007).

Despite similarities in environment, nordic skiing and alpine skiing are different in terms of terrain and skiing techniques. nordic skiing is practiced on flatter terrains with gently rolling undulating hills, and tracks are often narrow and grooved, while alpine terrains are steeper with sharp changes in direction. Therefore, the visual functions and levels of impairment affecting sports performance might differ between nordic and alpine skiing due to the differences in the visual tasks involved in these sports. Comparatively longer nordic skiing courses might require sustained visual and physical performance for a longer time compared to alpine skiing, which is completed in much shorter durations. However, the visual demands during the competitions could be higher in alpine skiing (albeit for short durations) due to the relatively higher speed involved in alpine skiing compared to nordic skiing (Erickson, 2007). Therefore, nordic and alpine skiing should be considered independently while investigating the sports-specific visual functions.

Preliminary studies conducted with Para nordic and Para alpine skiers using a test battery including a broad range of vision assessments such as static and dynamic VA, CS, low-contrast VA, glare sensitivity (GLS), glare recovery (GLR), and color vision reported that none of these visual functions were individually predictive of Para nordic skiing performance, while static VA was a significant predictor of Para alpine slalom performance (Creese et al., 2017a, c). However, measuring CS with the Pelli-Robson chart was not feasible in these populations due to the limitations in letter size as well as the spatial frequency range of the chart (Creese et al., 2017a, c). GLS and GLR could only be measured monocularly during these preliminary studies as well, and participants with a broad range of experience and skill participated, which could have confounded the true vision–performance relationship because of the variable impact of skill on performance (Creese et al., 2017a, b, c). Thus, it was concluded that future studies with refined test batteries are required to identify the visual functions associated with skiing performance (Creese et al., 2017a, b, c).

The purpose of the two independent prospective studies presented in this manuscript was to reexamine the relationship between vision and sport performance in elite, experienced Para nordic and Para alpine skiers of similar skill using a refined vision test battery. The inferences from these studies were also used to identify the visual functions that should be included in the classification systems for Para nordic and Para alpine skiing. It was hypothesized that CS, static VA, and visual field (VF) were associated with skiing performance.

## Materials and Methods

These studies used an observational research design and adhered with the tenets of the Declaration of Helsinki. All international level Para nordic and Para alpine skiers were given the opportunity to participate, and informed consent was obtained from all participants. This study was reviewed by and received ethics clearance through a University of Waterloo Research Ethics Committee.

### Participants

Elite Para nordic and Para alpine skiers were recruited with the help of the International Paralympic Committee (IPC) at the 2017 Para nordic World Championships (WCH, Finsterau, Germany), 2018 Para nordic World Cup (WC, Oberried, Germany), and the 2017 Para Alpine WCH (Tarvisio, Italy). A total of 26 Para nordic skiers (20 from WCH and 6 from WC) and 15 Para alpine skiers participated in the studies. WCH events are the most prestigious Para nordic and Para alpine events next to the Paralympic Games, with only the elite, most competitive skiers as participants. Recruiting from WCH events ensured that the study participants had comparable levels of skiing skill. The six additional Para nordic skiers recruited at the WC event were also eligible to participate in the WCHs but had not competed at the WCH event in 2017 for political reasons. For reference, there were only 42 Para nordic skiers (WCH, 2017 = 29; WC, 2018 = 13) and 23 Para alpine skiers who were WCH eligible and competing at the events included in this study, and there were only 46 Para nordic skiers and 34 Para alpine skiers in the world who were WCH eligible and registered with the IPC at the time of the study. Therefore, 61.9% of eligible Para nordic skiers and 65.2% of eligible Para alpine skiers participated in these studies, which accounted for 56.5 and 44.1% of the world’s entire population of elite Para nordic and Para alpine skiers, respectively. Although small, the study samples were representative of the elite Para nordic and Para alpine skiers’ populations.

### Procedure

Each participant in these studies attended a single study visit. During the study visit, participants completed a questionnaire (Supplementary Appendix A) about their skiing experience, which included questions about their vision impairment and skiing history as well as their current average annual training routine both on- and off-snow. Participants’ visual functions were assessed using a test battery that was determined based on the previous feasibility studies conducted by the research team (Creese et al., 2017a). The test battery included binocular tests of static VA, CS, GLS, GLR, light sensitivity (LS), dynamic VA, translational motion perception (TMP), radial motion perception (RMP), and VF. The participants’ performance on the visual function assessments was compared with their skiing performance (described below).

Static VA was measured using an Early Treatment Diabetic Retinopathy Study (ETDRS) chart at 1 m and/or the Berkeley Rudimentary Vision Test (BRVT) at 0.25 to 1 m with an external illumination on 395 ± 10% lux (Ferris et al., 1982). Standard measurement procedures with letter-by-letter scoring were incorporated during the static VA assessments using ETDRS (Klein et al., 1983; Ferris and Bailey, 1996). Participants started reading the letters at the top of the chart and continued to read down until they could no longer identify a minimum of three out of the five letters on a line correctly. To ensure that VA could be calculated at the borders near the limits of measurement of both charts (i.e., where both charts overlap), the single-letter BRVT tumbling E targets were each presented 5 times as there are 5 letters per line on the ETDRS charts, and letter-by-letter scoring was used (each letter correctly identified valued at 0.02 logMAR; Vanden Bosch and Wall, 1997). The ETDRS and BRVT charts were chosen to measure static VA in this study, because these charts used by the IPC for classification of athletes with vision impairments.

Contrast sensitivity was measured using the quick CSF (contrast sensitivity function) procedure on an Adaptive Sensory Technology platform (AST, Germany). The AST platform consisted of a 46″ NEC P463 screen with 1920 × 1080 resolution, calibrated to 90-cd/m^2^ background luminance. At a viewing distance of 1 m, the screen allowed a display of stimuli in a spatial frequency range of 0.35 to 9 cycles per degree. It was possible to present contrast levels down to 0.2% reliably (Dorr et al., 2017b). Three letters were presented horizontally during a trial with the left and middle letters displayed at four and two times the contrast of the right letter, respectively. A CSF was calculated after 25 trials. The area under the log CSF curve (AULCSF, logCS units) calculated by the software was used as the summary statistic for the CS assessments (Hou et al., 2010; Jia et al., 2014). AULCSF was chosen as the summary measure of CSF because it has been reported to have better predictive power and test–retest precision compared to peak CSF using fractional rank precision analyses (Dorr et al., 2017a, b).

A novel device (D&zzle, V&mp Vision Suite, University of Waterloo) was used to measure GLS and GLR. GLS was estimated by measuring the static VA of participants immediately after introducing a bright, binocular glare source in the line of sight. GLR was measured by retesting the static VA 1 minute after the glare source was removed. Static VA in the presence of and after removing the glare source was compared to the baseline static VA (no glare) to determine the GLS and GLR, respectively. LS was assessed by measuring the static VA of participants at increased light levels (approximately 1900 lux, both in the surround as well as on the chart). Static VA in the presence of the bright light was compared to the baseline static VA to determine LS in logMAR units. GLS was calculated using the following formula: *GLS* = *Static*VAin thepresence of*glare*−*StaticVA*. GLR and LS were also calculated similarly. Positive logMAR values for GLS, GLR, and LS indicated that VA worsened compared to baseline during the respective testing conditions.

Dynamic VA was measured using the computer program moV& (V&mp Vision Suite, Waterloo, Canada) with a single moving tumbling *E* letter that moved in a random walk trajectory at a speed of 1 m/s and was presented on a high definition television screen (50″ or 60″ display, 60 Hz refresh rate and 1920 × 1080 resolution, illuminance at 130–150 lx) at a distance of 1 m (Hirano et al., 2017). The initial size of the letter presented was 0.60 log units bigger than the participant’s static VA to make sure that the subject started the test from a suprathreshold level, and the maximum letter size presentable on this screen was 2.60 logMAR at a distance of 1 m. Five targets were presented per 0.1 logMAR step, and the display time was set to be unlimited to ensure adequate time to respond to the direction of the letter *E*. The test sequence continued until the participant could no longer correctly identify three out of five targets of the same size. Dynamic VA was also recorded in logMAR units, using a per letter scoring system (each letter correctly identified valued at 0.02 logMAR; Bailey et al., 1991).

Random dot kinematograms consisting of 100 individual, full-contrast, local dots that were equivalent to the size of the target detail of a 2.00 logMAR letter were used to assess two types of global motion tasks: translational (up and down) motion (TMP) and radial (in and out) motion (RMP; Dalton et al., 2017). Stimuli were presented on high-definition television screens (50″ or 60″ displays, 60 Hz refresh rate and 1920 × 1080 resolution, illuminance at 130–150 lx). On each TMP and RMP trial, the stimulus was presented for a maximum of 16 s, and participants were asked to identify the motion direction of the signal dots. The testing followed a staircase method, which was terminated after eight reversals, and the threshold was calculated by averaging the last six reversals.

**FIGURE 1:**
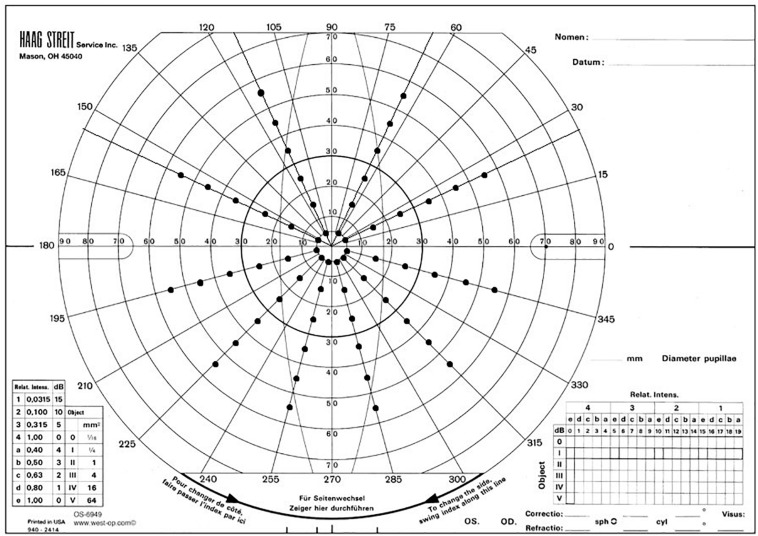
Modified AMA scoring grid on a Goldmann VF scoring sheet for functionally scoring VF. This figure was adapted from the unpublished report of Mann and Ravensbergen. Protocol for AMA-Style Analysis of Visual Field, 2019 (Mann and Ravensbergen, 2019).

A VF was binocularly assessed using an Arc perimeter and recorded following the standardized protocol, which was modified to allow binocular measurement (Grosvendor, 2007). VF assessments were performed by the same examiner (AS), moving a 6-mm-diameter target (size IV) from non-seeing areas (starting from 90° eccentricity) in the far periphery to the seeing areas at a speed of approximately 3–5 degrees per second. Once the VF boundary was identified, the target was moved continuously along each axis toward the central fixation point to identify any scotoma, if present. The edges of scotomata were reassessed until the response was consistent and reliable. Testing always started with the horizontal axis, and once the horizontal axis was marked, the arc was rotated to test the entire 360 degree VF in 30-degree intervals (Grosvendor, 2007). The current Paralympic classification uses Humphrey field analyzer, Octopus, and Goldman perimeters for assessing athletes’ VFs. However, it was not feasible to use one of these instruments in this study because the study locations were remote on the ski venues; therefore, the Arc perimeter was chosen because of its portability. The VF assessment method used during the data collection for this study was found to be reliable and valid when compared with the Humphrey field analyzer in a separate study (Stalin, 2020). An unbiased modified AMA scoring method (AMA 6E, Figure 1) designed by Mann and Ravensbergen was used for functionally scoring VF data of the nordic and alpine participants in the studies in order to ensure no assumptions were made about which aspects of the VF had more importance in skiing performance. The maximum possible VF score was 60, and the scores were converted to percentages [i.e., (AMA 6E score/60) ^∗^100] (Mann and Ravensbergen, 2019). The VF scoring method used in the study was also validated and compared with the Humphrey field analyzer (Stalin, 2020).

### Skiing Performance

Multiple confounding factors such as fatigue, jetlag, weather conditions, anxiety, or an illness could affect an individual’s skiing performance; therefore, we calculated the overall performance points for each participant based on the World Para nordic Skiing (WPNS) and World Para Alpine Skiing (WPAS) scoring systems rather than choosing a single race. The WPNS and WPAS scoring systems award skiers points, based on their best performances in a rolling validity period. In WPNS, skiers’ best five performances in a 24-month window are used to determine skiers’ performance points, while in WPAS, skiers’ best two performances in a 15-year period are used. The performance points in WPAS are discipline specific [downhill (DH), super G (SG), giant slalom (GS), and slalom (SL)], but not in WPNS.

In both Para nordic and Para alpine skiing, skiers with VI compete for one medal, regardless of their class. The WPNS and WPAS scoring systems adjust skiers’ race times by a class factor, such that skiers with most severe impairments receive a maximum time bonus. In order to ensure that the skiing performance metric was not impacted by skiers’ previous classification, skiers’ points across the season were recalculated without the class factor, so that performance was determined based on skiers’ raw times. Performance points calculated in these studies are referred to as raw-WPNS or raw-WPAS points to differentiate them from publicly available, published race results.

The formula for calculating unfactored race points was $P=\left(\left(\frac{T\,x}{T\,0}\right)-1\right)*F+\mathrm{race}\,\mathrm{pentalty}$, where *P* = race points, T_*X*_ = raw race time of competitor in seconds, T_0_ = raw race time of the overall gender best performer in seconds, and *F* = discipline factor (determined by IPC and reevaluated once in every 2 years based on competition results). The race penalty is another factor determined by the IPC to account for the quality of competition and ensures that race points from different competitions can be compared equitably. Using this formula, best skiers have the lowest performance points (World Para Nordic Skiing, 2018, 2019; World Para Alpine Skiing, 2019).

This formula calculates race points relative to the race time of the overall best performer in each race, for each gender. Previous research has also demonstrated that gender does not affect visual functions, such as VA or CS (Beck et al., 1993; Ohlsson and Villarreal, 2005; Grobbel et al., 2016). As performance points were normalized to the best performance in each gender and visual function does not appear to differ between genders, researchers were able to compare performance data between genders, which was important because of the small number of elite alpine and nordic skiers with vision impairment in the world.

For Para nordic skiing, the validity period for the calculation of raw-WPNS points used in this study was from 1 April 2016 to 31 March 2018. For Para alpine skiing, the validity period used was from 1 January 2016 to 31 March 2017. All skiers included in these studies completed the minimum number of races needed to calculate their raw-race points based on the sport rules (five for Para nordic and two for Para alpine). Not all Para alpine skiers in this study competed in each discipline, but all skiers included in the study completed at least two races in at least one discipline.

The use of other tests to quantify confounding factors related to skiing performance, such as visual motor reaction times under different physiological conditions (i.e., fatigue, anxiety) to measure attention, and tests of muscle strength and flexibility was considered and ultimately dismissed. The wide range of vision impairments in the study populations, including athletes with marked VF loss and athletes with no light perception (NLP), made visual motor reaction time testing unfeasible. Tests of muscle strength and flexibility would have certainly provided insight into athlete fitness levels, but male and female athletes would have likely performed differently on these tests and stratifying the performance analysis by gender would have further reduced our sample size and statistical power.

### Data Analysis

Data analysis (SPSS for Windows, version 25.0, SPSS, Inc.) focused on (1) determining the associations between skiing performance with vision-related and non-vision-related variables such as skiers’ age, age started skiing, age of onset of impairment, total lifetime hours of skiing, and number of races completed in the period that the skiing performance points were calculated for, and (2) identifying the visual function assessments, which could be predictive of skiing performances taking into account the non-vision variables. Based on the recommendations of the Joint Position Stand on Paralympic classification, correlation models and regression analyses were chosen to identify the visual functions that are predictive of sports performance and to compare the visual function measures and the performance measures (Mann and Ravensbergen, 2018). Kendall τ was chosen for correlation analysis as it guards against outliers among the marginal distributions and is reported to have smaller gross error sensitivity and asymptotic variance, making it more robust and efficient compared to the Spearman correlation (Croux and Dehon, 2010; Wilcox, 2016). Bonferroni–Holm corrections were used to account for multiple comparisons in the correlation analyses as it is a more powerful sequentially rejective multiple-testing approach that strongly controls the family-wise error rate compared to the traditional Bonferroni corrections (Holm, 1979; Simes, 1986). Considering the small sample sizes, including all 14 independent variables would have resulted in overfitting and increased variation inflation factors (VIF; Hawkins, 2004; Kim, 2019). Therefore, Kendall τ correlations were used as a guideline for choosing variables for multivariable regressions. Any variables that demonstrated significant (*p* < 0.05) or near-significant (*p* < 0.1) correlations with skiing performance were included in the multivariable regression models, conducted using the enter method. Multicollinearity and VIFs were assessed before finalizing the variables. There were no outliers in the data, and the assumptions of normality and homoscedasticity were met in both Para nordic and Para alpine data (Olive, 2017). In Para alpine, each discipline was analyzed separately.

Seven Para nordic and two Para alpine participants had light perception (LP) or NLP vision, and values of 3.8 logMAR and 4.2 logMAR were arbitrarily assigned for their static VA, respectively, so they could be included in the correlation and regression analyses on the same continuous scale as the other participants. Similarly, values of 0.00 log CS and 0.0% were assigned for these participants’ CS and VF measures, respectively. Dynamic VA has been reported to be between 0.20 and 0.30 logMAR worse than the static VA in individuals with normal vision (Hirano et al., 2017), but it was impossible to predict how much worse dynamic VA would be relative to static VA for each individual with vision impairment. Assigning 0.00 logMAR values for GLS, GLR, or LS for these participants would indicate that their static VA did not change with glare or increased light intensity rather than they were unable to do the task. Similarly, assigning a 100% value for their TMP and RMP would indicate that they were able to perceive the motion at 100% coherence, not that they were unable to do the task. Therefore, it was not appropriate to assign the same, or adjusted, arbitrary values for dynamic VA, GLS, GLR, LS, TMP, or RMP and no values were substituted for these visual function parameters in the Para nordic and Para alpine participants with LP or NLP vision.

*A priori* power analysis (G^∗^power 3.1.9.7) indicated that sample sizes of 85 would be required to obtain a minimum level of power of.80 (Cohen, 2013) with an alpha of.05 with medium effect size (0.15; Faul et al., 2007). However, it was impossible to recruit 85 skiers for each study as there were only 46 Para nordic and 34 Para alpine elite skiers with vision impairment in the world.

*Post hoc* power analysis (G^∗^power 3.1.9.7) indicated that the power to detect the obtained effects at the effect size of 0.15 and alpha error probability of 0.05 were 0.36 in the Para nordic, 0.20 in GS and SL, and 0.28 in SG for the regression analyses in prediction of skiing performance (Faul et al., 2007). These analyses suggest that the Para nordic and Para alpine studies did not have sufficient power to support the analysis results but considering the uniqueness of study population and the fairly representative samples in the studies, the results are still meaningful.

## Results

Twenty-six Para nordic skiers from 13 nations and 15 Para alpine skiers from 10 nations who competed at the events where testing took place participated in these studies (Table 1). Summary visual function data for each sport are found in Tables 2, 3. The arbitrarily assigned values for static VA, CS, and VF were not included in the calculation of means and standard deviations presented in these summary tables because they were not actual measured values of the participants. The arbitrary values were only included in the correlation and regression analyses.

**TABLE 1:** Participant details and summary statistics of their non-vision variables by sport.

	Para nordic	Para alpine
Number of athletes	26	15
Gender	18 Male; 8 female	8 Male; 7 female
Number of nations	13	10
Age (years)	26.0 ± 6.3	28.1 ± 11.6
Age range (years)	18 to 43	16 to 58
Age started skiing (years)	12.8 ± 8.2	16.2 ± 8.2
Age of onset of impairment (years)	6.8 ± 8.1	5.3 ± 7.1
Total lifetime hours of skiing	4545.5 ± 3883.5	4239.3 ± 4094.0
Number of races during the validity period	12.2 ± 4.9	DH: 6.8 ± 2.1 (*N* = 9)
		GS: 8.9 ± 3.4 (*N* = 15)
		SG: 7.4 ± 3.4 (*N* = 13)
		SL: 13.7 ± 5.0 (*N* = 15)

*The summary statistics presented in this table include the mean ± standard deviation of all the athletes.*

**TABLE 2:** Summary of visual function assessments of Para nordic skiing participants.

Visual function tests	*N*	Mean ± SD	Median	Range
Static visual acuity (logMAR)	19	1.71 ± 0.40	1.60	1.18 to 2.68
Contrast sensitivity (logCS)	19	0.21 ± 0.26	0.12	0.00 to 0.82
Glare sensitivity (change in logMAR)	19	0.20 ± 0.31	0.10	–0.19 to 0.98
Glare recovery (change in logMAR)	19	0.06 ± 0.20	0.00	–0.20 to 0.79
Light sensitivity change in logMAR)	19	0.00 ± 0.09	0.00	–0.15 to 0.16
Dynamic visual acuity (logMAR)	16	1.80 ± 0.31	1.80	1.20 to 2.20
Translational motion perception (%)	15	59.8 ± 26.9	61.8	19.2 to 100.0
Radial motion perception (%)	15	62.8 ± 28.5	61.2	26.5 to 100.0
Visual field (%)	19	63.9 ± 26.9	71.7	3.3 to 100.0

*Only the participants’ data with measurable results on each test are included. The data presented do not include participants with LP or NLP vision.*

**TABLE 3:** Summary of visual function assessments of Para alpine skiing participants.

Visual function tests	*N*	Mean ± SD	Median	Range
Static visual acuity (logMAR)	13	1.20 ± 0.51	1.40	0.04 to 1.64
Contrast sensitivity (logCS)	13	0.53 ± 0.59	0.40	0.00 to 1.90
Glare sensitivity (change in logMAR)	13	0.19 ± 0.17	0.14	0.02 to 0.54
Glare recovery (change in logMAR)	13	0.05 ± 0.08	0.02	–0.06 to 0.18
Light sensitivity change in logMAR)	10	0.09 ± 0.14	0.04	–0.08 to 0.34
Dynamic visual acuity (logMAR)	11	1.48 ± 0.57	1.40	0.50 to 2.20
Translational motion perception (%)	12	56.4 ± 31.9	53.3	9.3 to 100.0
Radial motion perception (%)	12	56.8 ± 29.0	55.3	12.8 to 100.0
Visual field (%)	13	53.5 ± 28.5	55.0	16.7 to 100.0

*Only the participants’ data with measurable results on each test are included. The data presented do not include participants with LP or NLP vision.*

Among the Para nordic participants, five had NLP and two had LP vision. Among the Para alpine participants, one had NLP and one had LP vision. One Para alpine participant also had very good static VA (-0.04 logMAR); however, this participant had qualified for the competition based on the extent of their VF (7.5°radius).

Both the Para nordic and Para alpine skiers had a broad range of ocular pathologies. Ocular diseases affecting the central retina, peripheral retina, and total retina were most common among Para nordic and Para alpine skiers. 62% of the Para nordic participants and 53% of Para alpine participants had onset of VI after age 2. Forty percent (40%) of the Para nordic and 63% of the Para alpine skiers had VI conditions that were progressive. The most common VF defect among both Para nordic and Para alpine skiers was a peripheral VF constriction. Further details on the types of VF defects can be found in Supplementary Appendix B.

The average raw-WPNS points of Para nordic participants was 58.73 ± 52.44 (range: 0.00 to 172.07, *N* = 26). The average raw-WPAS points of Para alpine participants for DH discipline was 155.81 ± 66.36 (range: 33.99 to 254.19, *N* = 9), GS was 226.98 ± 212.13 (range: 51.11 to 854.02, *N* = 15), SG was 336.20 ± 341.34 (range: 50.09 to 1299.41, *N* = 13), and SL was 193.40 ± 185.03 (range: 66.77 to 722.13, *N* = 15).

### Associations of Visual Functions and Skiing Performance

**FIGURE 2:**
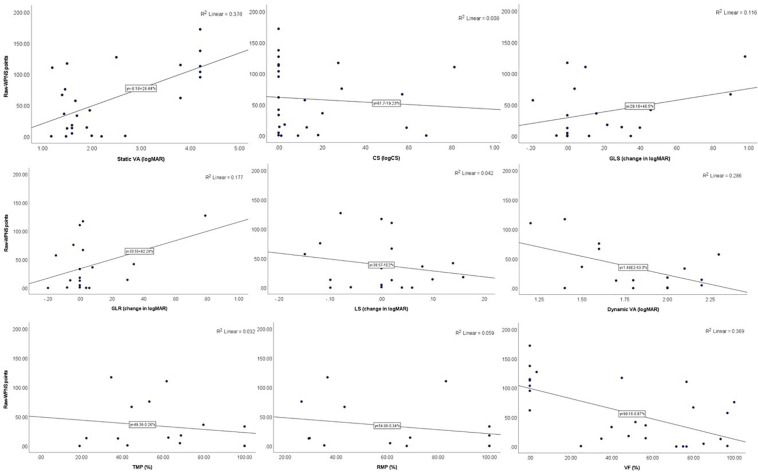
Scatter plots showing the relationships between raw-WPNS points and visual functions.

In Para nordic skiing, participants’ raw-WPNS points were significantly correlated with the AMA 6E scoring of VFs (*p* = 0.011). There were also trends toward significance for static VA (*p* = 0.066) being correlated with raw-WPNS points (Table 4 and Figure 2).

**TABLE 4:** Summary of correlations of visual functions with skiing performances; *p*-values are presented in the table with sample sizes, and significant correlations are provided in bolded text.

Variable	Raw-WPNS points	DH Raw-WPAS points	GS Raw-WPAS points	SG Raw-WPAS points	SL Raw-WPAS points
Static VA (logMAR)	*τ*_*b*_ = 0.26, *p* = 0.066 (26)	***τ*_*b*_ = 0.54, *p* = 0.046 (9)**	***τ*_*b*_ = 0.50, *p* = 0.010 (15)**	***τ*_*b*_ = 0.57, *p* = 0.007 (13)**	*τ*_*b*_ = 0.35, *p* = 0.074 (15)
CS (logCS)	*τ*_*b*_ = −0.23, *p* = 0.124 (26)	*τ*_*b*_ = −0.50, *p* = 0.061 (9)	***τ*_*b*_ = −0.46, *p* = 0.017 (15)**	***τ*_*b*_ = −0.51, *p* = 0.017 (13)**	*τ*_*b*_ = −0.37, *p* = 0.059 (15)
GLS (change in logMAR)	*τ*_*b*_ = 0.18, *p* = 0.301 (19)	*τ*_*b*_ = 0.31, *p* = 0.206 (9)	*τ*_*b*_ = 0.21, *p* = 0.357 (13)	*τ*_*b*_ = −0.02, *p* = 1.000 (11)	*τ*_*b*_ = 0.08, *p* = 0.759 (13)
GLR (change in logMAR)	*τ*_*b*_ = 0.21, *p* = 0.225 (19)	*τ*_*b*_ = 0.48, *p* = 0.075 (9)	*τ*_*b*_ = −0.01, *p* = 0.951 (13)	*τ*_*b*_ = −0.13, *p* = 0.583 (11)	*τ*_*b*_ = 0.12, *p* = 0.668 (13)
LS (change in logMAR)	*τ*_*b*_ = −0.06, *p* = 0.724 (19)	*τ*_*b*_ = −0.20, *p* = 0.543 (7)	*τ*_*b*_ = −0.21, *p* = 0.417 (10)	*τ*_*b*_ = −0.33, *p* = 0.262 (8)	*τ*_*b*_ = −0.16, *p* = 0.528 (10)
Dynamic VA (logMAR)	*τ*_*b*_ = −0.22, *p* = 0.238 (16)	*τ*_*b*_ = 0.59, *p* = 0.044 (8)	*τ*_*b*_ = 0.25, *p* = 0.306 (11)	*τ*_*b*_ = 0.46, *p* = 0.092 (9)	*τ*_*b*_ = −0.06, *p* = 0.813 (11)
TMP (%)	*τ*_*b*_ = −0.10, *p* = 0.728 (15)	*τ*_*b*_ = 0.44, *p* = 0.095 (9)	*τ*_*b*_ = 0.39, *p* = 0.084 (12)	***τ*_*b*_ = 0.49, *p* = 0.041 (11)**	*τ*_*b*_ = 0.23, *p* = 0.299 (12)
RMP (%)	*τ*_*b*_ = −0.24, *p* = 0.317 (15)	*τ*_*b*_ = 0.03, *p* = 0.917 (9)	*τ*_*b*_ = 0.08, *p* = 0.731 (12)	*τ*_*b*_ = −0.04, *p* = 0.876 (11)	*τ*_*b*_ = −0.02, *p* = 0.945 (12)
VF (%)	***τ*_*b*_ = −0.37, *p* = 0.011 (26)**	*τ*_*b*_ = 0.09, *p* = 0.753 (9)	*τ*_*b*_ = −0.33, *p* = 0.090 (15)	*τ*_*b*_ = −0.34, *p* = 0.110 (13)	***τ*_*b*_ = −0.49, *p* = 0.013 (15)**

**FIGURE 3:**
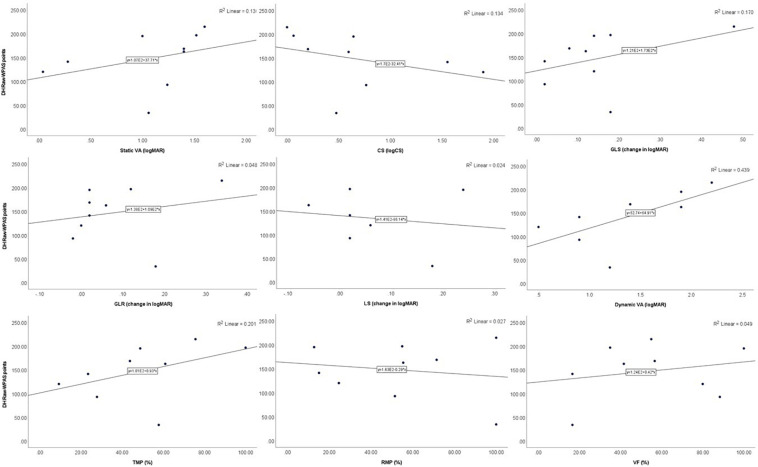
Scatter plots showing the relationships between DH raw-WPAS points and visual functions.

**FIGURE 4:**
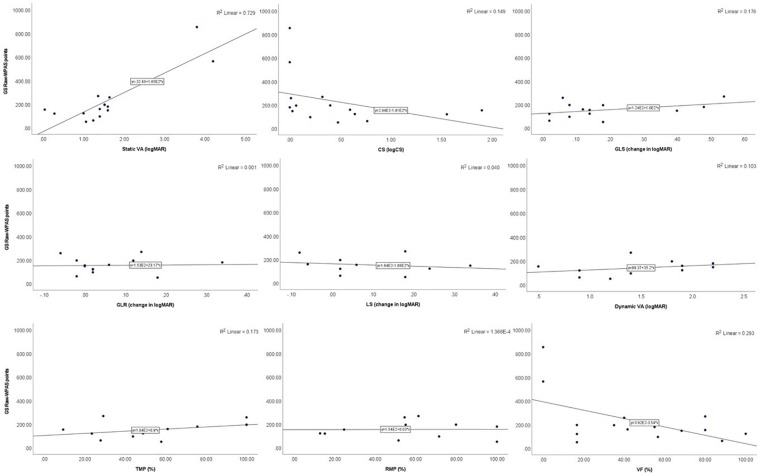
Scatter plots showing the relationships between GS raw-WPAS points and visual functions.

**FIGURE 5:**
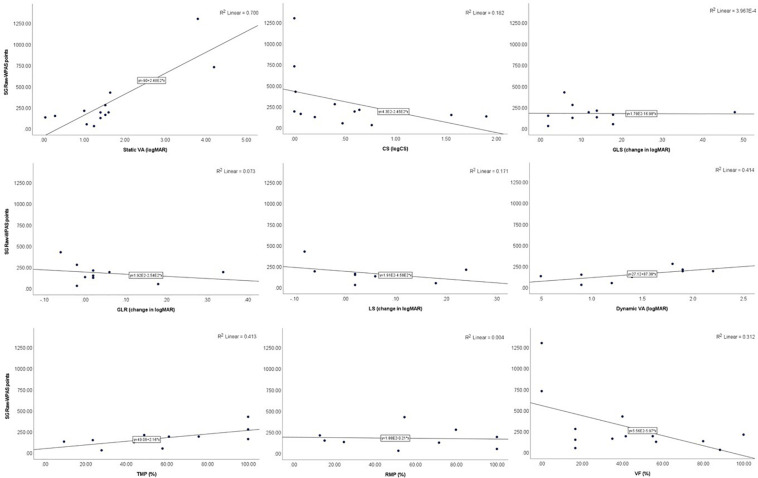
Scatter plots showing the relationships between SG raw-WPAS points and visual functions.

**FIGURE 6:**
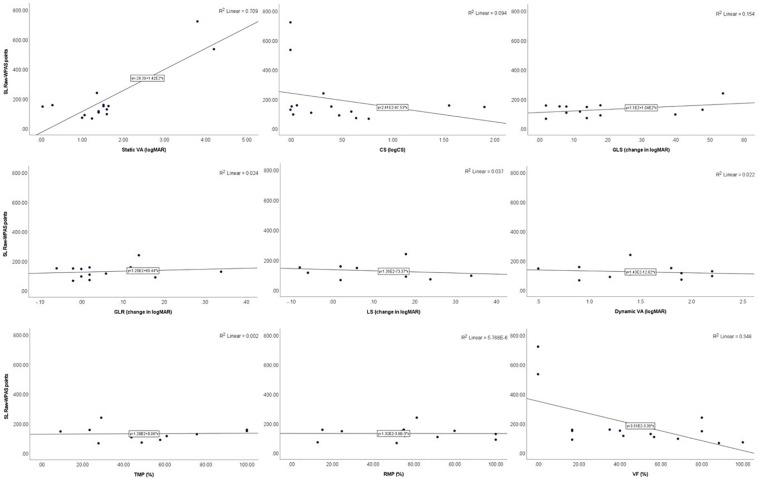
Scatter plots showing the relationships between SL raw-WPAS points and visual functions.

In Para alpine, static VA was significantly associated with raw-WPAS points in DH (*p* = 0.046), GS (*p* = 0.010), and SG (*p* = 0.007) and was nearly significant in the SL discipline (*p* = 0.074). TMP was significantly associated with raw-WPAS points in SG (*p* = 0.041) and VF was significantly associated with raw-WPAS points SL (*p* = 0.013). TMP also demonstrated a trend toward significance with raw-WPAS points in DH (*p* = 0.095) and GS (*p* = 0.084). CS was significantly associated with raw-WPAS points in GS (*p* = 0.017) and SG (*p* = 0.017) and was nearly significant in DH (*p* = 0.06) and SL (*p* = 0.06; Table 4 and Figures 3–6).

None of the correlations in the Para nordic or Para alpine data were significant after applying the Bonferroni–Holm correction. The summary of the correlation analyses of the vision-related variables with the adjusted, Bonferroni–Holm-corrected, *p*-values in the Para nordic and Para alpine data are provided in Table 5.

**TABLE 5:** Summary of correlations of visual functions with skiing performances.

Variable	Raw-WPNS points	DH Raw-WPAS points	GS Raw-WPAS points	SG Raw-WPAS points	SL Raw-WPAS points
Static VA (logMAR)	*τ*_*b*_ = 0.26, *p* = 0.792 (26)	*τ*_*b*_ = 0.54, *p* = 0.598 (9)	*τ*_*b*_ = 0.50, *p* = 0.140 (15)	*τ*_*b*_ = 0.57, *p* = 0.098 (13)	*τ*_*b*_ = 0.35, *p* = 0.77 (15)
CS (logCS)	*τ*_*b*_ = −0.23, *p* = 1.000 (26)	*τ*_*b*_ = −0.50, *p* = 0.72 (9)	*τ*_*b*_ = −0.46, *p* = 0.221 (15)	*τ*_*b*_ = −0.51, *p* = 0.221 (13)	*τ*_*b*_ = −0.37, *p* = 0.708 (15)
GLS (change in logMAR)	*τ*_*b*_ = 0.18, *p* = 1.000 (19)	*τ*_*b*_ = 0.31, *p* = 1.000 (9)	*τ*_*b*_ = 0.21, *p* = 1.000 (13)	*τ*_*b*_ = −0.02, *p* = 1.000 (11)	*τ*_*b*_ = 0.08, *p* = 1.000 (13)
GLR (change in logMAR)	*τ*_*b*_ = 0.21, *p* = 1.000 (19)	*τ*_*b*_ = 0.48, *p* = 1.000 (9)	*τ*_*b*_ = −0.01, *p* = 1.000 (13)	*τ*_*b*_ = −0.13, *p* = 1.000 (11)	*τ*_*b*_ = 0.12, *p* = 1.000 (13)
LS (change in logMAR)	*τ*_*b*_ = −0.06, *p* = 1.000 (19)	*τ*_*b*_ = −0.20, *p* = 1.000 (7)	*τ*_*b*_ = −0.21, *p* = 1.000 (10)	*τ*_*b*_ = −0.33, *p* = 1.000 (8)	*τ*_*b*_ = −0.16, *p* = 1.000 (10)
Dynamic VA (logMAR)	*τ*_*b*_ = −0.22, *p* = 1.000 (16)	*τ*_*b*_ = 0.59, *p* = 0.616 (8)	*τ*_*b*_ = 0.25, *p* = 1.000 (11)	*τ*_*b*_ = 0.46, *p* = 0.90 (9)	*τ*_*b*_ = −0.06, *p* = 1.000 (11)
TMP (%)	*τ*_*b*_ = −0.10, *p* = 1.000 (15)	*τ*_*b*_ = 0.44, *p* = 1.000 (9)	*τ*_*b*_ = 0.39, *p* = 0.96 (12)	*τ*_*b*_ = 0.49, *p* = 0.492 (11)	*τ*_*b*_ = 0.23, *p* = 1.000 (12)
RMP (%)	*τ*_*b*_ = −0.24, *p* = 1.000 (15)	*τ*_*b*_ = 0.03, *p* = 1.000 (9)	*τ*_*b*_ = 0.08, *p* = 1.000 (12)	*τ*_*b*_ = −0.04, *p* = 1.000 (11)	*τ*_*b*_ = −0.02, *p* = 1.000 (12)
VF (%)	*τ*_*b*_ = −0.37, *p* = 0.169 (26)	*τ*_*b*_ = 0.09, *p* = 1.000 (9)	*τ*_*b*_ = −0.33, *p* = 1.000 (15)	*τ*_*b*_ = −0.34, *p* = 1.000 (13)	*τ*_*b*_ = −0.49, *p* = 0.182 (15)

*The adjusted *p*-values based on the Bonferroni–Holm corrections are presented in the table with sample sizes.*

### Associations of Non-Vision-Related Variables and Skiing Performance

Participants’ number of races during the point calculation period was significantly correlated with the raw-WPNS points (*p* = 0.010) and SG raw-WPAS points (*p* = 0.031). Total hours of skiing in lifetime was also nearly significantly correlated (*p* = 0.098) with raw-WPNS points (Table 6). None of the correlations in the Para nordic or Para alpine data were significant after applying the Bonferroni–Holm correction (Table 7).

**TABLE 6:** Summary of correlations of non-vision variables with skiing performances.

Variable	Raw-WPNS points (*N* = 26)	DH Raw-WPAS points (*N* = 9)	GS Raw-WPAS points (*N* = 15)	SG Raw-WPAS points (*N* = 13)	SL Raw-WPAS points (*N* = 15)
Age (years)	*τ*_*b*_ = 0.05, *p* = 0.707	*τ*_*b*_ = 0.06, *p* = 0.833	*τ*_*b*_ = 0.17, *p* = 0.371	*τ*_*b*_ = 0.12, *p* = 0.581	***τ*_*b*_ = 0.39, *p* = 0.047**
Age started skiing (years)	*τ*_*b*_ = 0.19, *p* = 0.422	*τ*_*b*_ = 0.31, *p* = 0.249	*τ*_*b*_ = 0.17, *p* = 0.371	*τ*_*b*_ = 0.03, *p* = 0.903	*τ*_*b*_ = 0.31, *p* = 0.112
Age of onset of impairment (years)	*τ*_*b*_ = −0.06, *p* = 0.657	*τ*_*b*_ = −0.03, *p* = 0.914	*τ*_*b*_ = −0.17, *p* = 0.411	*τ*_*b*_ = −0.28, *p* = 0.218	*τ*_*b*_ = 0.08, *p* = 0.681
Total hours of skiing	*τ*_*b*_ = −0.23, *p* = 0.098	*τ*_*b*_ = −0.06, *p* = 0.835	*τ*_*b*_ = −0.11, *p* = 0.586	*τ*_*b*_ = −0.18, *p* = 0.393	*τ*_*b*_ = 0.03, *p* = 0.882
Number of races	***τ*_*b*_ = −0.37, *p* = 0.010**	*τ*_*b*_ = −0.12, *p* = 0.669	*τ*_*b*_ = −0.19, *p* = 0.343	***τ*_*b*_ = −0.46, *p* = 0.031**	*τ*_*b*_ = −0.17, *p* = 0.371

*The unadjusted *p*-values are presented in the table with sample sizes, and significant correlations are provided in bolded text.*

**TABLE 7:** Summary of correlations of non-vision variables with skiing performances.

Variable	Raw-WPNS points (*N* = 26)	DH Raw-WPAS points (*N* = 9)	GS Raw-WPAS points (*N* = 15)	SG Raw-WPAS points (*N* = 13)	SL Raw-WPAS points (*N* = 15)
Age (years)	*τ*_*b*_ = 0.05, *p* = 1.000	*τ*_*b*_ = 0.06, *p* = 1.000	*τ*_*b*_ = 0.17, *p* = 1.000	*τ*_*b*_ = 0.12, *p* = 1.000	*τ*_*b*_ = 0.39, *p* = 0.611
Age started skiing (years)	*τ*_*b*_ = 0.19, *p* = 1.000	*τ*_*b*_ = 0.31, *p* = 1.000	*τ*_*b*_ = 0.17, *p* = 1.000	*τ*_*b*_ = 0.03, *p* = 1.000	*τ*_*b*_ = 0.31, *p* = 1.000
Age of onset of impairment (years)	*τ*_*b*_ = −0.06, *p* = 1.000	*τ*_*b*_ = −0.03, *p* = 1.000	*τ*_*b*_ = −0.17, *p* = 1.000	*τ*_*b*_ = −0.28, *p* = 1.000	*τ*_*b*_ = 0.08, *p* = 1.000
Total hours of skiing	*τ*_*b*_ = −0.23, *p* = 1.000	*τ*_*b*_ = −0.06, *p* = 1.000	*τ*_*b*_ = −0.11, *p* = 1.000	*τ*_*b*_ = −0.18, *p* = 1.000	*τ*_*b*_ = 0.03, *p* = 1.000
Number of races	*τ*_*b*_ = −0.37, *p* = 0.130	*τ*_*b*_ = −0.12, *p* = 1.000	*τ*_*b*_ = −0.19, *p* = 1.000	*τ*_*b*_ = −0.46, *p* = 0.403	*τ*_*b*_ = −0.17, *p* = 1.000

*The adjusted *p*-values based on the Bonferroni–Holm corrections are presented in the table with sample sizes. None of the values retained significance after applying the Bonferroni–Holm corrections.*

### Associations Between Visual Functions and Non-Vision Related Variables

In the Para nordic study population, one of the non-vision variables—number of races—had significant correlations with static VA (*τ*_*b*_ = -0.45, *p* = 0.002) and VF (*τ*_*b*_ = 0.314, *p* = 0.032). None of the other non-vision variables had significant correlations with any of the other visual functions in the Para nordic study population (*p* > 0.05). There were no significant correlations between any of the visual functions and non-vision variables in the Para alpine study population (*p* > 0.05).

### Visual Functions Predictive of Skiing Performances

Multivariable regression analysis was used to look at whether or not skiing performances could be predicted based on any of the individual visual functions measured. Static VA and VF were the only two visual function variables that demonstrated strong enough correlations (*p* < 0.1) with Para nordic skiing performance to be considered in the model. Based on the correlation analyses, static VA, VF, number of races, and total hours of skiing were included in the modeling for Para nordic skiing performance, and a significant regression equation was found *F*(4,21) = 7.12, *p* = 0.001, and *R*^2^ = 0.58. Para nordic predicted raw-WPNS points were equal to 130.484 – 3.981 (number of races) – 0.006 (Total hours of skiing; Stalin et al., 2019). In other words, a participant’s Para nordic skiing performance improved by 3.981 points for each race competed by the participant during the points calculation period and by 0.006 points for each hour of skiing.

Static VA, dynamic VA, CS, TMP, and VF showed a significant association or were nearly significantly associated with performance in one or more of the Para alpine disciplines and were considered for inclusion in the multivariable regression. Static VA had strong significant correlations with skiing performances in most of the Para alpine disciplines, and static VA was also strongly correlated with dynamic VA, CS, and TMP, likely due to the wide range of VI among participants in the study populations (Dalton et al., 2019; Stalin et al., 2019). Including all these variables in modeling resulted in multicollinearity and high VIF (Kim, 2019). To avoid the instability and the overfitting due to multicollinearity in the multivariable regression model, static VA was chosen for inclusion in the model as static VA had the strongest correlations overall with each of the other visual functions and skiing performance. Thus, the final regression model for Para alpine included static VA, VF, skier’s age, and the number of races.

There was no significant regression equation for DH [*F*(4,4) = 0.46, *p* = 0.76, and *R*^2^ = 0.32]. For GS, a significant regression equation was found *F*(4,10) = 14.36, *p* < 0.001, and *R*^2^ = 0.85. Para alpine participants’ predicted GS raw-WPAS points were equal to -74.472 + 166.991 (static VA) +5.557 (age), where static VA was measured in logMAR units and age was measured in years. Participants’ Para alpine GS skiing performance points deteriorated by 166.991 points for each 1.00 logMAR increase in static VA (worsening) and by 5.557 points for an increase in each year of age.

For SG, a significant regression equation was found *F*(4,8) = 8.71, *p* = 0.05, and *R*^2^ = 0.81. Para alpine participants’ predicted SG raw-WPAS points were equal to 13.714 + 217.007 (static VA), where static VA was measured in logMAR units. Participants’ Para alpine SG skiing performance points deteriorated by 217.007 points for each 1.00 logMAR increase in static VA.

Similar to the GS results, a significant regression equation was found *F*(4,10) = 14.66, *p* < 0.01, and *R*^2^ = 0.85 for SL performance points. Para alpine participants’ predicted SL raw-WPAS points were equal to -164.532 + 145.066 (static VA) +5.739 (age), where static VA was measured in logMAR units and age was measured in years. Participants’ Para alpine SL skiing performance deteriorated by 145.066 points for each 1.00 logMAR increase in static VA and by 5.739 points for an increase in each year of age.

## Discussion

These studies were conducted to identify visual functions associated with, and predictive of, Para nordic, and Para alpine skiing performance. Ideally, large study populations would have been recruited to assess the significance of a broad range of vision functions on the skiing performance, as was done in these studies, because the high variation in the other non-vision factors could mask the effects of vision on performance. To ensure that the variations in non-vision factors such as skill development, training, and coaching levels were as small as possible between the participants, only elite skiers were recruited for these studies. Additionally, in recognition that the sample sizes were small, robust statistical analysis methods such as Kendall τ correlations were conducted and Bonferroni–Holm *post hoc* adjustments were done. *Post hoc* power analyses suggested that these studies did not have the power to support the study results due to the limited sample sizes. However, the Para nordic and Para alpine skiers’ populations in the world are unique and small, making it impossible to obtain large sample populations for the studies. These studies recruited approximately half of the entire world’s World Championship eligible Para nordic and Para alpine populations registered to compete at the time of the study and over 60% of the athletes in each sport who were competing at the events where testing took place. Therefore, despite the small sample sizes, it can be seen that the study populations in these studies were very representative of the populations of Para nordic and Para alpine skiers with vision impairment.

While various competition events within Para nordic skiing sport (e.g., sprint, middle distance, and long distance) differ mainly in terms of only the length of the courses, disciplines within the Para alpine skiing sport differ in terms of the terrain characteristics and the skiing techniques used. As a result, these Para alpine disciplines also differ in terms of the skiers’ participation. Slalom and GS are the two most popular Para alpine disciplines, with participation from skiers with the most severe impairments (B1 class). DH had the least number of participants and rarely has participation from skiers in the B1 class, probably due to the increased speed (maximum speed of 150 km/h) and high visual demands involved in DH (Gilgien et al., 2018). Additionally, DH courses are steeper and longer compared to the courses used in other alpine disciplines, which limits their availability to some athletes for training. The limited availability of well-groomed DH courses for training might also have reflected in reduced participation. It is also reported that DH is the alpine discipline that is reported to have the highest injury incidence rates (Flørenes et al., 2011). These variations in skiers’ participation were reflected in the Para alpine population participated in this study as well.

The results of the Para nordic study suggested that even though static VA had possible association and VF had strong association with Para nordic skiing performance, neither individual vision function was predictive of Para nordic skiing performance. Multiple factors such as training, skill development, and coaching levels influence the performance of skiers in addition to various physical and psychological factors, which are unique to each individual. Participants were chosen from an elite population to specifically minimize the impact of variations in such non-vision factors on the skiing performance of the study populations. However, even within such an elite Para nordic study population, the only predictive factors of skiing performance were the number of races that the participants competed in and skiers’ total lifetime hours. Therefore, the training variables seemed to have a more significant impact on Para nordic skiing performance than static VA and VF. None of the other vision variables, including CS, seemed to be associated with Para nordic skiing performance, contrary to expectations.

Consistent with the previous reports on the visual demands associated with alpine skiing, performance in the Para alpine technical disciplines, which have participation from skiers with a wide range of VI, seemed to be predicted by the static VA and associated with VF when the age of skiers was taken into account (Craybiel et al., 1955; Senner et al., 1999). Better static VA was predictive of better GS, SG, and SL performance points, which require more technical skill and less speed than DH. Similarly, better CS also appeared to be associated with, though not predictive of, skiers’ performance in the Para alpine skiing technical disciplines. Though not predictive, better VF was also associated with better SL performances. Therefore, static VA and CS appeared to be associated with the performance of Para alpine skiers in all the disciplines except in DH. Slalom is the most technical alpine discipline, requiring athletes sometimes to look several gates ahead and shift their gaze frequently between different gate positions (Decroix et al., 2017). The need to attend to multiple gates might be the reason behind the association of VF with the SL performance. Better performance in the DH discipline, which requires more speed, was associated with better dynamic VA. TMP, RMP, GLS, GLR, and LS were not significantly associated with performance in either sport.

Static VA is one of the most common assessments of spatial vision and is extremely useful to detect deficits in the visual system. Although the static VA does not seem to directly provide information about the perception of low-contrast images or objects in motion, previous research had reported significant strong correlations between the static VA and measures of CS and dynamic VA (Burg and Hulbert, 1961; Burg, 1966; Lesmes et al., 2012). Consistent with previous literature, static VA of both Para nordic and Para alpine skiers was significantly strongly associated with dynamic VA and CS in current studies (Dalton et al., 2019; Stalin et al., 2019) and similar to those reported in low vision populations, especially when CS was measured using the qCSF method (Lesmes et al., 2012; Stellmann et al., 2015). Correlations between CS and static VA have been shown to be the strongest in heterogeneous populations, such as the population of athletes with diverse types of vision impairments studied here (Haegerstrom-Portnoy et al., 2000). Thus, it is possible that the strong correlations between static VA and CS found in this population of skiers could have masked the relationships between CS and skiing performance in these studies.

In addition to the small study sample size, these studies had some limitations. Depth perception has the potential to be one of the vision functions associated with skiing performance; however, due to the lack of a feasible instrument to measure distance stereopsis in low vision populations, it was not possible to include an assessment for depth perception in the test battery. Para nordic and Para alpine skiers also wear a wide range of ski goggles when competing for protection from external elements such as wind and glare. The impact of these ski goggles on the Para skiers’ performance is currently not known, but none of the glare or light sensitivity metrics (GLS, GLR, or LS) measured in the absence of tints and filters were significantly associated with skiing performance. As the use of tints and filters could affect various aspects of vision including CS and glare, future studies could explore the effect of using different tints or filters in the skiing sports.

The test battery for the study was chosen based on consideration of multiple factors such as the test availability, portability, current sport classification rules, and each test’s precision and accuracy. While we used the qCSF, which generated automated CSFs with high precision, traditional manual methods were used for measuring the static VA and VF. The current Paralympic classification rules rely on printed charts (ETDRS and BRVT) to assess static VA and standard automated perimetry to assess VF. Printed charts are more prone to random and systematic errors (i.e., effect of inter/intra observer changes in instructions, termination rules, etc.; Rosser et al., 2003). However, the charts used in these studies are used by the sports for classification purposes, so the tradeoff in precision and accuracy is balanced with the increased utility for the sports. Data collection was all done at the ski venues which were in remote locations away from hospitals and optometric clinics in larger city centers. Therefore, an Arc perimeter was used for measuring the peripheral VFs in the studies as it was not feasible to use standard automated perimeters due to their lack of availability at the study locations and the limited portability of these instruments. The Arc perimeter was portable, was easy to use, and has been shown to be reasonably accurate when compared to a Humphrey automated perimeter to determine areas of seeing vs. non-seeing, as long as measurements are conducted by a single, trained researcher (Stalin, 2020).

Novel assessment methods such as the qVA (Adaptive Sensory Technology, Inc.) and qVFM (Adaptive Sensory Technology, Inc.) are potential tests that could be used in similar future studies. The qVA (Lesmes and Dorr, 2019; Zhao et al., 2021) and qVFM (Xu et al., 2019, 2020) are based on the Bayesian active learning and Bayesian adaptive method, respectively, and were reported to be high in precision and accuracy. However, the adoption of new tests would need to be carefully considered as tests included in classification need to be accessible globally and language independent so they can accommodate athletes from countries all over the world.

A modified AMA scoring system was used in these studies for functionally scoring the VFs of participants, to ensure no prior assumptions were made about which aspects of the VF were most important for skiing performance. Using this modified scoring system means the VF related results from these studies might not be directly comparable to future studies using different scoring methods, but the VF scoring system used here is being used consistently across all Paralympic classification research, so at least the results presented here can be compared to other Paralympic sports with vision impairments.

Finally, an ideal outcome measure for these studies would have been the raw race times of all participants on the same nordic or alpine course (depending on their discipline), under the same environmental and experimental conditions. The practicality issues involved in getting all these skiers from around the world to ski a single course on the same day made the ideal outcome measure impossible to achieve. However, choosing race times from a particular race instead of calculating the race performance points over a period of time as was done in this study may have increased the impact of confounding factors such as fatigue, poor weather, or anxiety on individual skiers performances. The point system used in this study ensured that the best performances of each elite skier in the validity period were considered for calculating the outcome measure, minimizing the effect of the abovementioned confounding factors. In addition, point calculations were based on the International Ski Federation (FIS) formula, which calculates race points relative to the race time of the overall best performer in each race, for each gender. Normalizing the performance points to the best performance in each gender allowed the researchers to compare performance data between genders. While additional tests to assess each skier’s physiological and psychological factors, such as attention and physical fitness, which could affect skiing performance were considered, ultimately, they were not included. Visual motor reaction time assessments of attention have limited utility in a population with diverse vision impairments and stratifying the population by muscle power and/or flexibility would have made comparison between genders more difficult, thereby reducing the study sample size. However, quantifying these confounding factors would have increased the validity of this study and should be considered for inclusion in similar future studies (Tweedy et al., 2014).

## Conclusion

In consideration of the correlation and regression analyses from these studies, static VA and VF were the only visual functions associated with both Para nordic and Para alpine skiing performance. Even though CS was associated with the performance in SG, GS, and SL disciplines and dynamic VA was associated with performance in DH, CS, and dynamic VA which were also strongly associated with static VA, CS, and dynamic VA do not appear to add any additional information for classification of skiers’ performance in either Para nordic or Para alpine skiing. From a Paralympic classification research point of view, a test should only be incorporated into classification if its addition to the test battery improves the ability of a classification system to minimize the impact of impairments on the outcome of a competition (Mann and Ravensbergen, 2018). Thus, these studies concluded that static VA and VF should be included as visual functions in Para nordic and Para alpine classification. Further research needs to be done in order to determine if other visual functions should also be included in classification.

## Data Availability Statement

The datasets presented in this article are not readily available because the data was collected in collaboration with the International Paralympic Committee and some aspects of the data set cannot be shared broadly. Please contact the corresponding author for more details if interested in the data. Requests to access the datasets should be directed to KD (kristine.dalton@uwaterloo.ca).

## Ethics Statement

The studies involving human participants were reviewed and approved by The Office of Research Ethics, University of Waterloo. The patients/participants provided their written informed consent to participate in this study.

## Author Contributions

The studies were designed by KD, and the data were collected by KD, MC, and AS. AS organized the database and performed the statistical analysis and interpretation presented in this manuscript. AS wrote the manuscript and KD revised, read, and approved the submitted version. All authors contributed to the article and approved the submitted version.

## Conflict of Interest

MC is currently employed by the International Paralympic Committee, and any views/conclusions expressed in the manuscript are her own and do not represent the views/conclusions of the International Paralympic Committee. The remaining authors declare that the research was conducted in the absence of any commercial or financial relationships that could be construed as a potential conflict of interest.
