# Supplementary material for: Do Impairments in Visual Functions Affect Skiing Performance?
Source: Front Neurosci. 2021 May 13;15:648648. doi: 10.3389/fnins.2021.648648 (PMC8155621; doi:10.3389/fnins.2021.648648)
Supplement: Supplementary file 1 [file Data_Sheet_1.docx]

1. *: Developmental History Questionnaire*

GENERAL PARTICIPANT INFORMATION

Participant ID #: ___________________________________________________

Age: ___________________________________________

Gender: _________________________________________________________

Sport: ___________________________________________________________

Competition events: _______________________________________________

Your vision impairment

At what age were you first diagnosed? ________________________________

*How did your impairment develop?*

Fully blind from birth

Vision impaired from birth and stable since

Vision impaired from birth, which progressively got worse over time:

Times of major progression were at ages ______ and ______ and ______

Vision impairment acquired at age _______ and stable since

Vision impairment acquired at age _______ , which progressively got worse over time:

Times of major progression were at ages ______ and ______ and ______

*Do you have any other impairments (e.g. a hearing impairment)?*

No

Yes, namely _____________________________________________________

YOUR VISION CORRECTION

Do you wear glasses? ___________________________________________________

Do you wear glasses while training? _________________________________

Do you wear glasses while competing? _______________________________

Are you wearing your glasses right now? _____________________________

Do you wear contacts lenses? ____________________________________________

Do you wear contacts while training? ________________________________

Do you wear contacts while competing? ______________________________

Are you wearing your contacts right now? ___________________________

**Your SKIING career**

In this section we wish to know more about your involvement in skiing.

*Is skiing your main sport? ___________________________________________________________________*

*Did you compete in other sports? If yes, at what ages? _______________________________________________*

|  | **AGE** | | | | | | | | | | | | | | | | | | | | | | | | | | | | | | |
| --- | --- | --- | --- | --- | --- | --- | --- | --- | --- | --- | --- | --- | --- | --- | --- | --- | --- | --- | --- | --- | --- | --- | --- | --- | --- | --- | --- | --- | --- | --- | --- |
|  | 5 | 6 | 7 | 8 | 9 | 10 | 11 | 12 | 13 | 14 | 15 | 16 | 17 | 18 | 19 | 20 | 21 | 22 | 23 | 24 | 25 | 26 | 27 | 28 | 29 | 30 | 31 | 32 | 33 | 34 | 35 |
| ***Training environment*** | | | | | | | | | | | | | | | | | | | | | | | | | | | | | | | |
| Local club |  |  |  |  |  |  |  |  |  |  |  |  |  |  |  |  |  |  |  |  |  |  |  |  |  |  |  |  |  |  |  |
| Regional training centre |  |  |  |  |  |  |  |  |  |  |  |  |  |  |  |  |  |  |  |  |  |  |  |  |  |  |  |  |  |  |  |
| State training centre |  |  |  |  |  |  |  |  |  |  |  |  |  |  |  |  |  |  |  |  |  |  |  |  |  |  |  |  |  |  |  |
| National training centre |  |  |  |  |  |  |  |  |  |  |  |  |  |  |  |  |  |  |  |  |  |  |  |  |  |  |  |  |  |  |  |
| ***Competition*** | | | | | | | | | | | | | | | | | | | | | | | | | | | | | | | |
| Regular competition |  |  |  |  |  |  |  |  |  |  |  |  |  |  |  |  |  |  |  |  |  |  |  |  |  |  |  |  |  |  |  |
| VI (Paralympic) competition |  |  |  |  |  |  |  |  |  |  |  |  |  |  |  |  |  |  |  |  |  |  |  |  |  |  |  |  |  |  |  |
| ***Sport class*** | | | | | | | | | | | | | | | | | | | | | | | | | | | | | | | |
| B3 |  |  |  |  |  |  |  |  |  |  |  |  |  |  |  |  |  |  |  |  |  |  |  |  |  |  |  |  |  |  |  |
| B2 |  |  |  |  |  |  |  |  |  |  |  |  |  |  |  |  |  |  |  |  |  |  |  |  |  |  |  |  |  |  |  |
| B1 |  |  |  |  |  |  |  |  |  |  |  |  |  |  |  |  |  |  |  |  |  |  |  |  |  |  |  |  |  |  |  |
| ***Vision impairment*** | | | | | | | | | | | | | | | | | | | | | | | | | | | | | | | |
| Stable |  |  |  |  |  |  |  |  |  |  |  |  |  |  |  |  |  |  |  |  |  |  |  |  |  |  |  |  |  |  |  |
| Progressing |  |  |  |  |  |  |  |  |  |  |  |  |  |  |  |  |  |  |  |  |  |  |  |  |  |  |  |  |  |  |  |

*Do you still compete in those sports? ____________________________________________________________*

Please use this first table to highlight (by colouring in the appropriate boxes in the table) the following:

- The ages at which you were practicing in different training environments (e.g. local club, regional training centre, state training centre, national training centre). There can of course be overlap in these. If you never trained in a certain environment, please just leave that blank.
- At what ages you competed in non-Paralympic competitions and when you competed in Paralympic competition.
- When you competed in the different VI sport classes (B3, B2, and B1).
- When your vision impairment was stable and when it was progressively getting worse.

With this next table we want to get some more detail about your training activities over the years. We are interested in how many hours you trained in an **average week** each year of your skiing career. Please split up your activities for each of your training environments and start from your current age and work backwards in time.


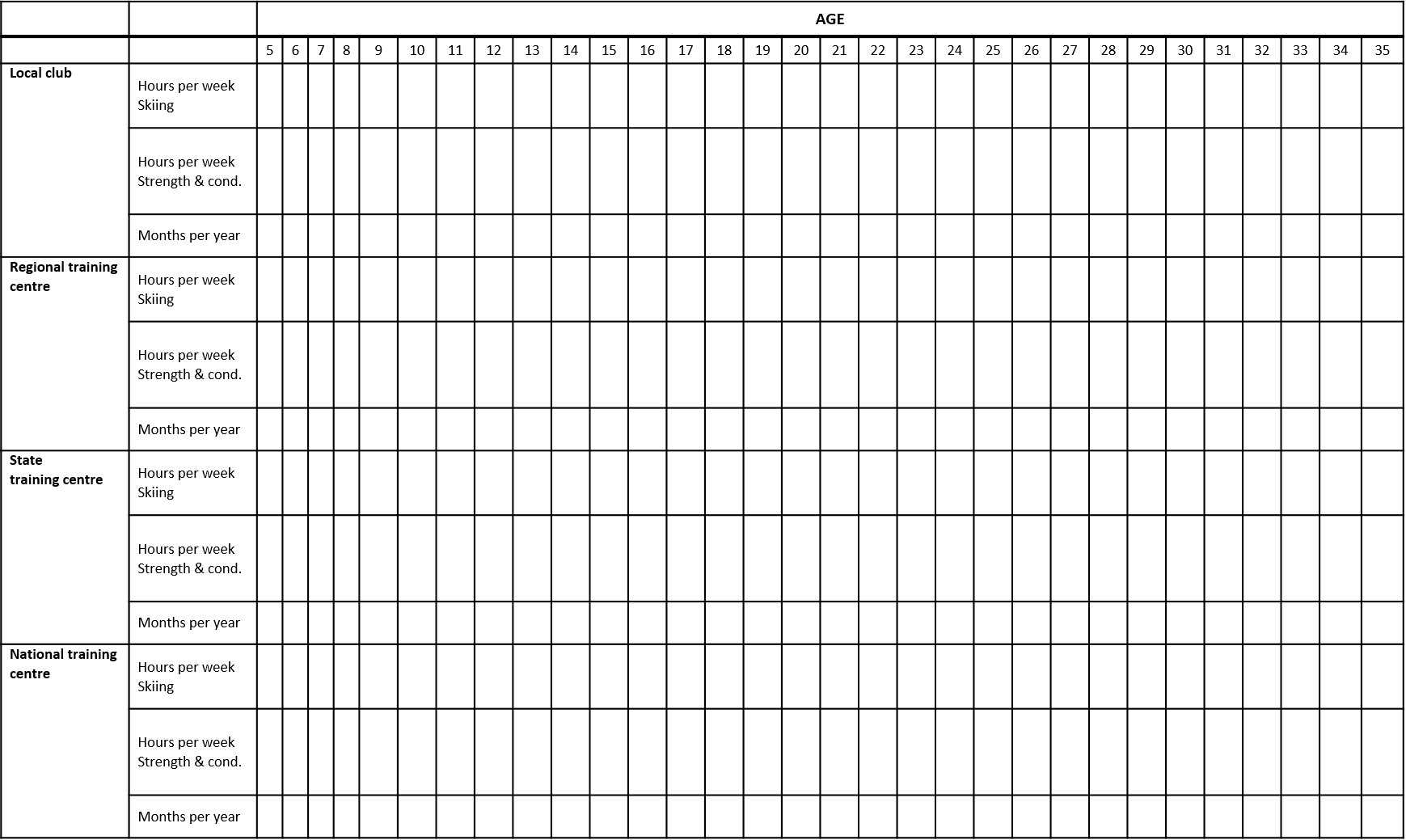


1. : *Ocular pathology diagnoses and visual field defects among Para nordic and Para alpine participants*

Both the Para nordic and Para alpine skiers had a broad range of ocular pathologies (Table B1). Ocular diseases affecting the central retina, peripheral retina, and total retina were most common among Para nordic and Para alpine skiers. 62% of the Para nordic participants and 53% of Para alpine participants had onset of VI after age 2. Forty percent (40%) of the Para nordic and 63% of the Para alpine skiers had VI conditions that were progressive. The most common VF defect among both Para nordic and Para alpine skiers was a peripheral VF constriction. Further details on the types of VF defects can be found in Table B2.

Table B1: Summary of ocular pathology diagnoses of Para nordic and Para alpine participants.

| **Ocular Pathology** | **Para nordic (N=26)** | **Para alpine (N=15)** |
| --- | --- | --- |
| Central Retina | Stargardt’s disease (2), macular degeneration (1), viteliform macular dystrophy (1), central retinal degeneration (2) | Stargardt’s disease (2), macular degeneration (1), achromatopsia (2) |
| Peripheral Retina | X linked retinoschisis (1), Leber's congenital amaurosis (2), retinitis pigmentosa (1), exudative genetic retinopathy (1), vitreo-choreo-retinal peripheral degeneration (1), peripheral retinal degeneration (1) | Retinitis pigmentosa (3) |
| Optic Nerve | Congenital optic nerve pathology (5) | Congenital coloboma of optic nerve (1), optic nerve atrophy (1) |
| Total Retina | Central and peripheral retinal dystrophy (2), retinal abiotrophy (1) | Retinopathy of prematurity (1), retinal dysplasia (1), retinal detachment (1), retinoblastoma (1) |
| Anterior Segment | Mature cataract (1), aphakia (1) |  |
| Total Globe | Microphthalmos (1), albinism (1), trauma (1) | Microphthalmos (1) |

Table B2: Summary of types of VF defects among Para nordic and Para alpine participants.

| **Type of visual field defect** | **Para nordic (N=26)** | **Para alpine (N=15)** |
| --- | --- | --- |
| Peripheral VF defect without central scotoma | 80.8% | 60.0% |
| Peripheral VF defect with scattered peripheral scotomata | 7.7% | Nil |
| Peripheral VF defect with central scotomata | 7.7% | 20.0% |
| Peripheral VF defect with ring scotomata | Nil | 6.7% |
| Tunnel vision (<10^0^ radius) | Nil | 13.3% |
| Peripheral island of vision | 3.8% | Nil |
